# Reply to: “Breast RT microcalcifications: a stress test for trainee-led magnetic-seed lumpectomy”

**DOI:** 10.1016/j.breast.2026.104793

**Published:** 2026-04-30

**Authors:** Fabio Corsi

**Affiliations:** a Department of Biomedical and Clinical Sciences, University of Milan, Milan, Italy; b Istituti Clinici Scientifici Maugeri IRCCS, Pavia, Italy

Dear Editor,

We sincerely thank Zhang et al. for their interest in our article [1]. Their observation about the higher rate of involves margins when microcalcifications are excised by residents under the guidance of magnetic seed warrants further considerations.

While intraoperative specimen radiography was performed in all cases as a standard clinical practice, cavity shave of margins was not performed in 24.5% of patients in the magnetic seed group. We agree with the Colleagues that cavity shave of margins should be considered an essential step of breast-conserving surgery, as previously reported in our experience [2]. Routine cavity shave might be even more relevant for non palpable lesions such as microcalcifications, especially for trainees. Defined supervision checkpoints are another important issue in this setting, since a step-by-step controlled approach (including check of specimen X-ray and completion of cavity shave) would ensure a standardized training avoiding heterogeneity in surgical practice.

About the second point, the calculated resection ratio (CRR) is already based on real 3D volumes (of both resected specimen and lesion), measured from the 3 dimensions obtained during surgical pathology procedures [3]. Since CRR is a ratio between the real excised volume and the “optimal” resection volume, we do not expect significant variations in our findings. Conversely, we agree that normalization according to type of oncoplastic reshaping would be greatly helpful, but it could not be performed on a retrospective series. Of course, it will be necessarily considered in further prospective studies.

We thank Zhang and Colleagues for the opportunity to better clarify these points of our study.

## Funding statement

No specific grant from any funding agency in the public, commercial, or not-for-profit sectors was received for this work.

## Declaration of competing interest

The author declares that they have no known competing financial interests or personal relationships that could have appeared to influence the work reported in this paper.
